# Identification of epithelial and mesenchymal circulating tumor cells in clonal lineage of an aggressive prostate cancer case

**DOI:** 10.1038/s41698-022-00289-1

**Published:** 2022-06-21

**Authors:** Shoujie Chai, Carmen Ruiz-Velasco, Amin Naghdloo, Milind Pore, Mohan Singh, Nicholas Matsumoto, Anand Kolatkar, Liya Xu, Stephanie Shishido, Ana Aparicio, Amado J. Zurita, James Hicks, Peter Kuhn

**Affiliations:** 1 Convergent Science Institute in Cancer, Michelson Center for Convergent Bioscience, University of Southern California, Los Angeles, CA 90089 USA; 2 Molecular and Computational Biology, Department of Biological Sciences, University of Southern California, Los Angeles, CA 90089 USA; 3 Department of Genitourinary Medical Oncology, University of Texas MD Anderson Cancer Center, Houston, TX 77030 USA

**Keywords:** Prostate cancer, Prognostic markers, Translational research, Tumour heterogeneity

## Abstract

Little is known about the complexity and plasticity of circulating tumor cell (CTC) biology in different compartments of the fluid microenvironment during tumor metastasis. Here we integrated phenomics, genomics, and targeted proteomics to characterize CTC phenotypic and genotypic heterogeneity in paired peripheral blood (PB) and bone marrow aspirate (BMA) from a metastatic prostate cancer patient following the rapid disease progression, using the High-Definition Single Cell Assay 3.0 (HDSCA3.0). Uniquely, we identified a subgroup of genetically clonal CTCs that acquired a mesenchymal-like state and its presence was significantly associated with one subclone that emerged along the clonal lineage. Higher CTC abundance and phenotypic diversity were observed in the BMA than PB and differences in genomic alterations were also identified between the two compartments demonstrating spatial heterogeneity. Single cell copy number profiling further detected clonal heterogeneity within clusters of CTCs (also known as microemboli or aggregates) as well as phenotypic variations by targeted proteomics. Overall, these results identify epithelial and mesenchymal CTCs in the clonal lineage of an aggressive prostate cancer case and also demonstrate a single cell multi-omic approach to deconvolute the heterogeneity and association of CTC phenotype and genotype in multi-medium liquid biopsies of metastatic prostate cancer.

## Introduction

The mutation, selection, and adaptation of tumor cells along the pathway of disease progression and metastasis results in a spectrum of phenotypic and genomic heterogeneity^1–4^. Distinguishing phenotypic states of tumor cells with genetically clonal identity and along clonal lineage is important in the investigation of epithelial-mesenchymal transition (EMT) and for understanding the link between genotype and phenotype^5,6^. Dissecting the genetic mechanism of a phenotypic transition is especially critical in circulating tumor cells (CTCs) with acquired metastatic capability as the key hallmark of cancer^7^. The underlying concept behind EMT is that a malignant epithelial cell changes its phenotypic state to become more mesenchymal-like and motile as a means to increase metastatic potential. This implies that to clearly identify EMT as a state change, the genomic alterations in the original epithelial cell must be evident in the resultant mesenchymal cell. Current liquid biopsy methods for CTC detection usually rely on one particular phenotypic state, traditionally the epithelial state characterized by cytokeratin and/or EpCAM expression^8^. Next generation approaches utilize enrichment-free methods such as the third generation of the High-Definition Single Cell Assay (HDSCA3.0) which has the ability to identify epithelial, mesenchymal, and endothelial phenotypic states among circulating rare cells, as well as to identify those cells in the genetically transformed tumor lineage in the liquid biopsy^9^.

A number of studies have shown that the presence of mesenchymal-like CTCs (mes.CTCs) is associated with worse prognosis^10–19^. However, the identification of mesenchymal CTCs in those studies was limited to immunostaining^11,13–16,19^, RT-PCR^10,17^, or RNA fluorescence in situ hybridization (RNA FISH)^12,18^ of EMT biomarkers, without further genomic validation of a genetic lineage with cancer cell identity. In our previous study^9^, of peripheral blood (PB) and bone marrow aspirates (BMA) from 65 metastatic castration-resistant prostate cancer patients in the “*cabazitaxel with or without carboplatin*” trial (NCT01505868)^20^, we identified a large number epithelial-like (CK+) cells that exhibited clonal genomic alterations characteristic of prostate cancer cells as well as platelet-coated cells that comprised a biomarker for therapeutic benefit from additional carboplatin. In that study, we also identified a small number of mesenchymal-like (CK+| VIM+) cells, however with the exception of two cases, they were genomically normal and thus may represent cells from the tumor microenvironment (TME) rather than tumor cells that had been transformed through EMT. Thus, dual identification of phenotypic states and genomic alterations on the same single cell is an essential approach to deconvolute EMT heterogeneity of CTCs with cancer cell identity confirmation and lineage tracing.

Among the potential patients for that trial, there was a single patient that underwent pre-enrollment evaluation but did not enter the trial due to rapid disease progression. Blood and bone specimens were taken as part of the pre-enrollment process and were thus available for this study. Here, we utilized the multi-omic capabilities of the HDSCA3.0 workflow to characterize the CTC phenotypes in paired PB and BMA samples from this unusually aggressive case, followed by single cell copy number profiling based on low pass whole genome sequencing or targeted proteomics based on imaging mass cytometry. In contrast to the patients in our previous study^9^, this index patient had large numbers of genetically clonal epithelial-like (CK+) CTCs and a nearly equal number of mesenchymal-like (CK+| Vim+) CTCs that comprised a genetic subclone of the original genomic alterations. Meanwhile, differences of CTC abundance and phenotypic diversity were observed between PB and BMA as well as genomic variations. The observed significant change in phenotypic state while maintaining clear genetic relationship to the transformed epithelial clone provides the clear evidence of the EMT state change detected in the liquid biopsy and further suggests that a multi-omic approach can provide useful information about patient condition in near real-time to influence treatment decisions.

## Results

### Patient demographics and HDSCA3.0 workflow

**Fig. 1:**
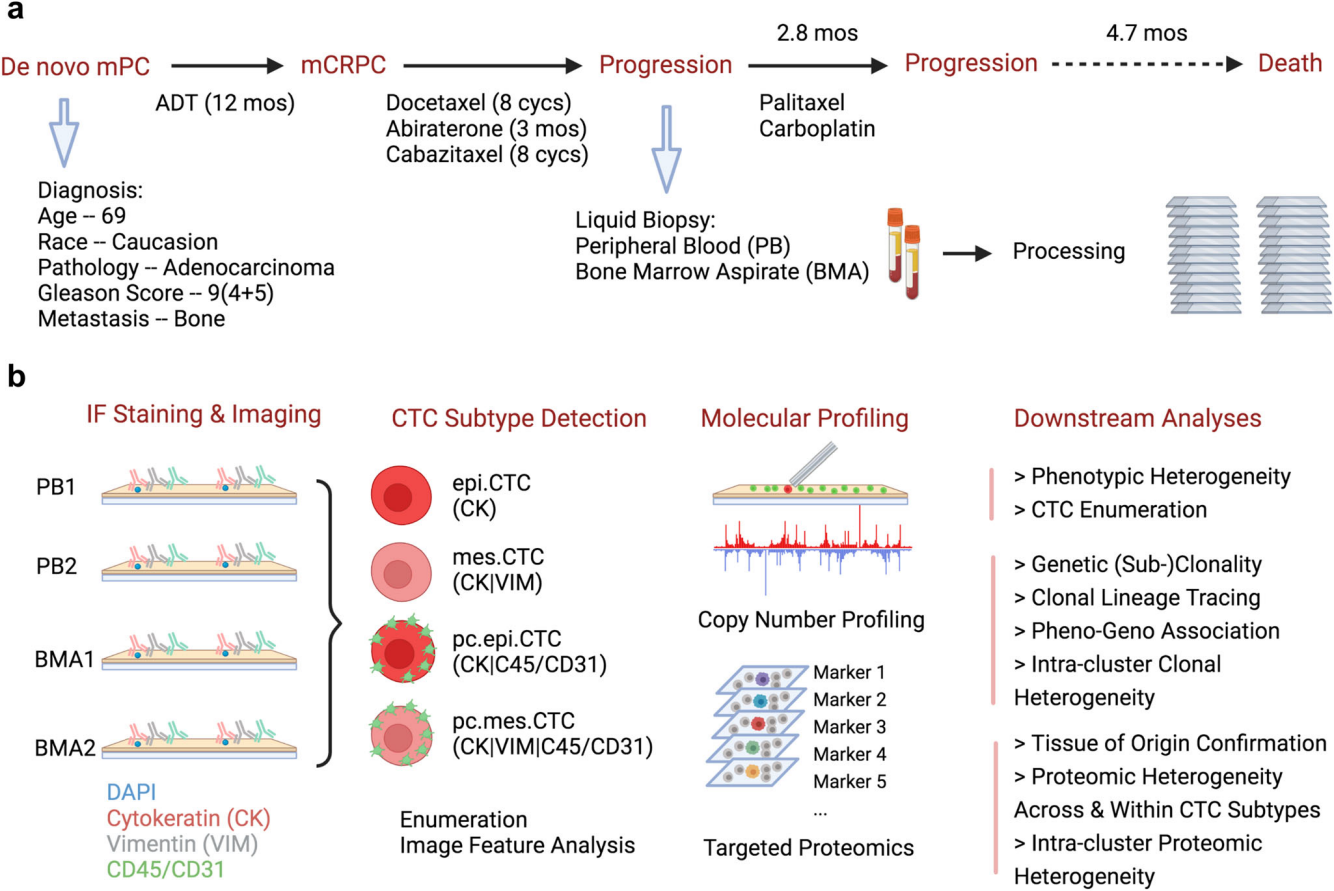
Patient demographic, pathology, and HDSCA3.0 workflow. **a** Patient’s diagnosis, pathology, disease progression, treatment history, and liquid biopsy timepoint. **b** HDSCA3.0 workflow including immunofluorescence staining and imaging, rare cell detection, CTC classification, molecular profiling, and downstream analyses.

The index patient was diagnosed with de novo mPC at age 69 with high-volume prostatic adenocarcinoma, Gleason Score 9 (4 + 5), PSA 66.4 ng/mL, and bone metastasis. He had acquired castrate resistance after only 12 months of androgen deprivation therapy and was subsequently treated with sequential treatments of docetaxel (8 cycles), abiraterone (3 months), and cabazitaxel (8 cycles). Following PSA progression (from 0.7 to 61.1 ng/mL within 2 months) from cabazitaxel therapy, paired PB and BMA were collected for HDSCA3.0 analysis before the 3rd line chemotherapy paclitaxel and carboplatin which maintained progression-free state for 2.8 months. Unfortunately, the patient passed away in 4.7 months after this progression. (Fig. 1a)

Two slides each from PB and BMA were processed with four-channel staining assay for CTC detection and phenotypic characterization. Furthermore, single cell copy number profiling by whole genome sequencing or targeted proteomics by image mass cytometry were utilized on selected CTCs for genotype and tissue of origin analysis. (Fig. 1b)

### CTC enumeration and phenotypical characterization

**Fig. 2:**
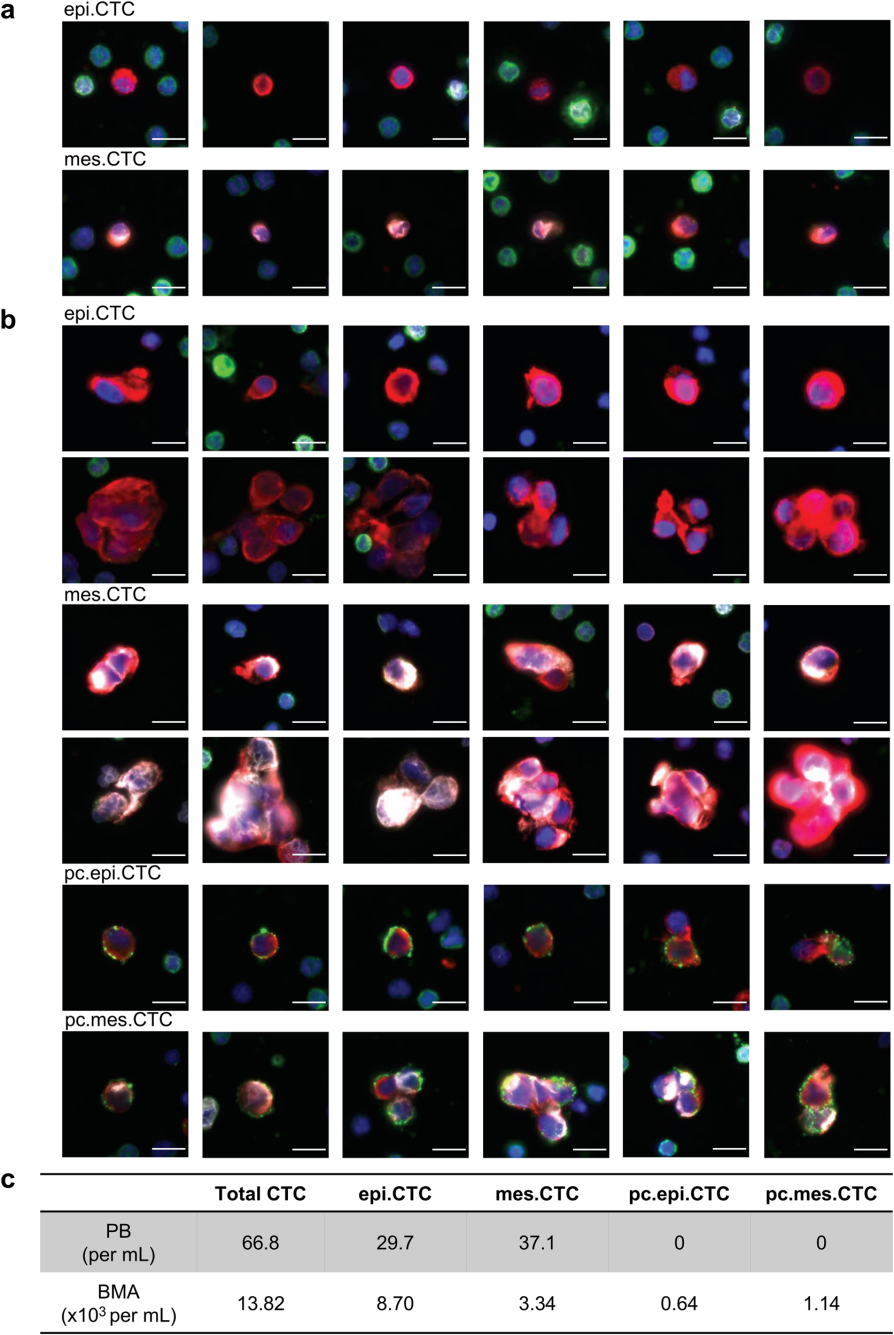
CTC subtype and enumeration in the paired PB and BMA. **a** Immunofluorescence images of CTCs from PB sample. Scale bar: 10 μm. **b** Immunofluorescence images of CTCs from BMA sample. Color coding: DAPI (blue); CK (red); VIM (white); CD45/CD31 (green). Scale bar: 10 μm. **c** Enumeration of CTC subtypes in each test of PB and BMA.

CTC subtypes were classified based on mesenchymal features and platelet attachment status, including epithelial-like CTCs (epi.CTCs), mes.CTCs, platelet-coated epithelial-like CTCs (pc.epi.CTCs), and platelet-coated mesenchymal-like CTCs (pc.mes.CTCs). Consistent with our previous publication^6,9,21^, the concentration of total CTCs in BMA was ~200-fold higher than in PB (13.82 × 10^3^ vs 66.8 cells per mL). Meanwhile a significant diversity of CTC phenotype was observed, particularly in BMA, with high incidence of CTC clusters and platelet attachment to CTCs compared to the PB (Fig. 2a–b & Supplementary Fig. 1). Within the BMA, 32.4% (4.48 × 10^3^ cells per mL) of CTC expressed VIM and 12.9% (1.78 × 10^3^ cells per mL) of them were coated with platelets. Interestingly, the fraction of VIM positivity was higher in the platelet-coated group, compared to non-coated cells (64.0% vs 27.8%) (Fig. 2c).

Based on EBImage-generated features from the four different fluorescence channels, we further evaluated intensity and morphological variations across four CTC subtypes. Following the feature selection pipeline (Supplementary Fig. 2a), we firstly identified ten unique image feature groups and further selected one feature per group as representatives, including four intensity features of DAPI, CK, VIM and CD45/CD31 and six morphological features, i.e., cell and nucleus sizes, cell and nucleus eccentricities, cell/nucleus size ratio, and nucleus location in cell (Supplementary Fig. 2b). As shown in Supplementary Fig. 3, there were minimal differences observed in morphological features, DAPI, or CK intensities among CTC subtypes except for the VIM and CD45/CD31 intensities.

### Clonal lineage and its variation between PB and BMA

**Fig. 3:**
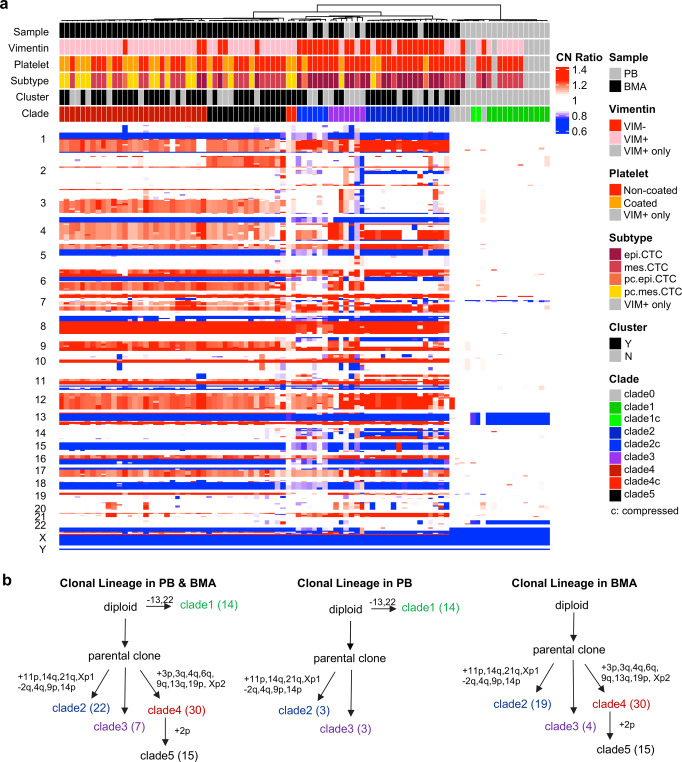
Clonal lineage and its variation between PB and BMA. **a** Complex heatmap of single cell copy number profiles from paired PB and BMA grouped by hierarchical clustering. The “c” in clade IDs means compressed profile due to normal cell contamination. Red, copy number gain; blue, copy number loss; white, copy number neutral. VIM + only refers to “VIM + only” rare cells. **b** Clonal lineage in PB and BMA combination and separate. The number in parenthesis is the number of cells analyzed.

Within PB and BMA samples, 93 cells were sequenced for single cell copy number profiling, including 85 CTCs (27 epi.CTCs, 33 mes.CTCs, 5 pc.epi.CTCs, 20 pc.mes.CTCs) and 8 “VIM + only” rare cells. Of all cells, 88 (94.6%) cells presented clonal alterations and further hierarchical clustering identified one main clone with multiple subclones (clade2–5, 74 cells) and one minor clone (clade1, 14 cells) (Fig. 3a). Unlike common copy number profiling of prostate cancer cells^22^, the minor clone (clade1) had only chromosome 13 and 22 losses and its breakpoints of chromosome 13 loss were different from those in the main clone (Supplementary Fig. 4c). Clonal lineage tracing indicated that the parental cell of the main clone divided into three different subclones (clade2, clade3, and clade4/5) with the acquisition of different CNAs and clade 5 was generated from clade4 with the additional chromosome 2p gain (Fig. 3b*left*) which was further confirmed by phylogenetic tree analysis of individual cells and subclones (Supplementary Fig. 5a–b). Intrasample comparison showed that the subclone (clade4/5) was uniquely detected in the BMA while the emerging minor clone (clade1) was only seen in the PB (Fig. 3b*mid & right*). As for the fraction of each subclone, 45 of 74 (60.8%) cells were from the clade4-5 (clade4: 30 cells, clade 5:15 cells), 22 cells were from clade2, and 7 cells were from clade3. Due to the small size and high heterogeneity of clade3, we mainly compare the differences between clade2 and clade4-5 and identified 17 distinct CNAs with numerous oncogenes and tumor suppressor genes involved (Supplementary Fig. 4a), e.g., 3q gain (PIK3CA, SOX2), 4q gain (PDGFR), 6q gain (ROS1), and 9q gain (NOTCH1) in the clade4-5 and 2q loss (ERCC3, ZEB2), 9p loss (JAK2, CDKN2A), and 14q gain (HSP90) in the clade2. There was no difference of LSTs observed between clade2 and clade4/5 (Supplementary Fig. 4b).

### Association between genomic subclone and mesenchymal phenotype

**Fig. 4:**
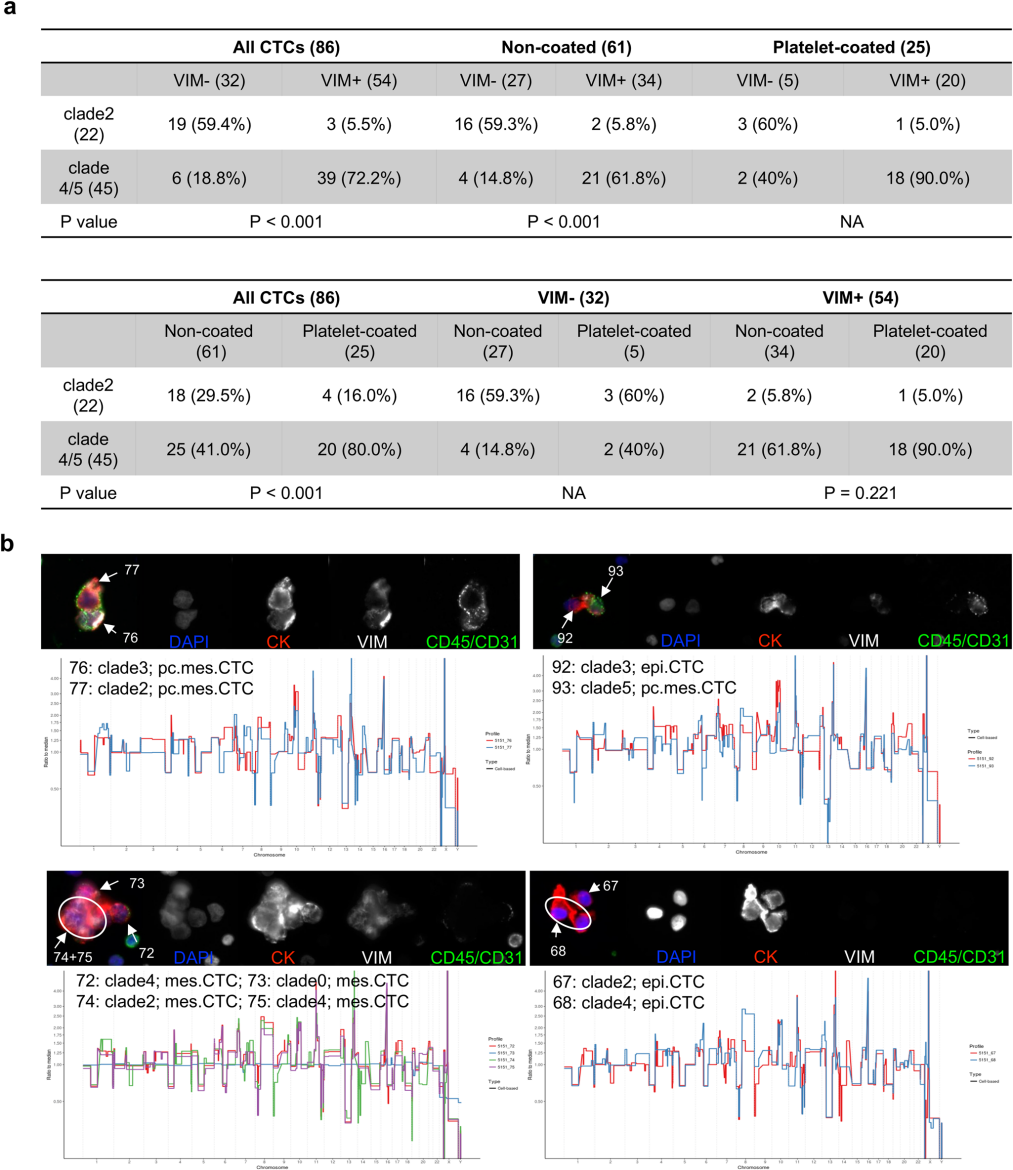
Association between genotype and phenotype. **a** Association of genomic subclones with mesenchymal phenotype or platelet attachment in all CTCs or subpopulations. **b** Immunofluorescence images and single cell copy number profiles of representative heterogeneous CTC clusters in the BMA sample. Images order: composite, DAPI, CK, VIM, and CD45/CD31. The number is the sequencing cell ID.

To characterize the relationship between the phenotype and the genotype of the CTCs from the index patient, we firstly compared fluorescence intensity between subclones and observed that the clades4/5 showed higher expression of VIM than clade2 (Supplementary Fig. 6a). To further test if phenotype could be more directly related to the genotype, we initially compared the fractions of clades 2/4/5 between VIM + and VIM− groups in all, non-coated and platelet-coated population. Results showed higher fraction of clade4/5 (39/54, 72.2%) observed in VIM + group while higher fraction of clade2 (19/32, 59.4%) in the VIM- group independent of platelet status, despite the small sample size in the platelet-coated population (Fig. 4a). Meanwhile, we examined the clade fraction difference between non-coated and platelet-coated groups as well and there was no significant difference in the VIM + population while the difference in the CTC population was present due to the sample size imbalance in VIM- population (Fig. 4a). The associations of VIM positivity, platelet attachment, CTC subtypes, and cluster status with genomic clonality were further visualized by t-SNE plots with the input of copy number data (Supplementary Fig. 6b).

Meanwhile, we investigated the association between subclones and cluster phenotype to see if genotypes of cells from the same cluster are heterogeneous or homogeneous. Among 14 CTC clusters sequenced from the BMA sample, 10 of them (71.4%) were homogeneous within the same clusters (3 clusters from clade2, 5 clusters from clade4, and 2 clusters from clade 5) and the other 4 (28.6%) clusters were heterogeneous, including various combinations, i.e., clade2 and 4, clade2 and 3, and clade3 and 5 (Fig. 4b & Supplementary Fig. 7a–b).

### Proteomic characterization of CTC subtypes

**Fig. 5:**
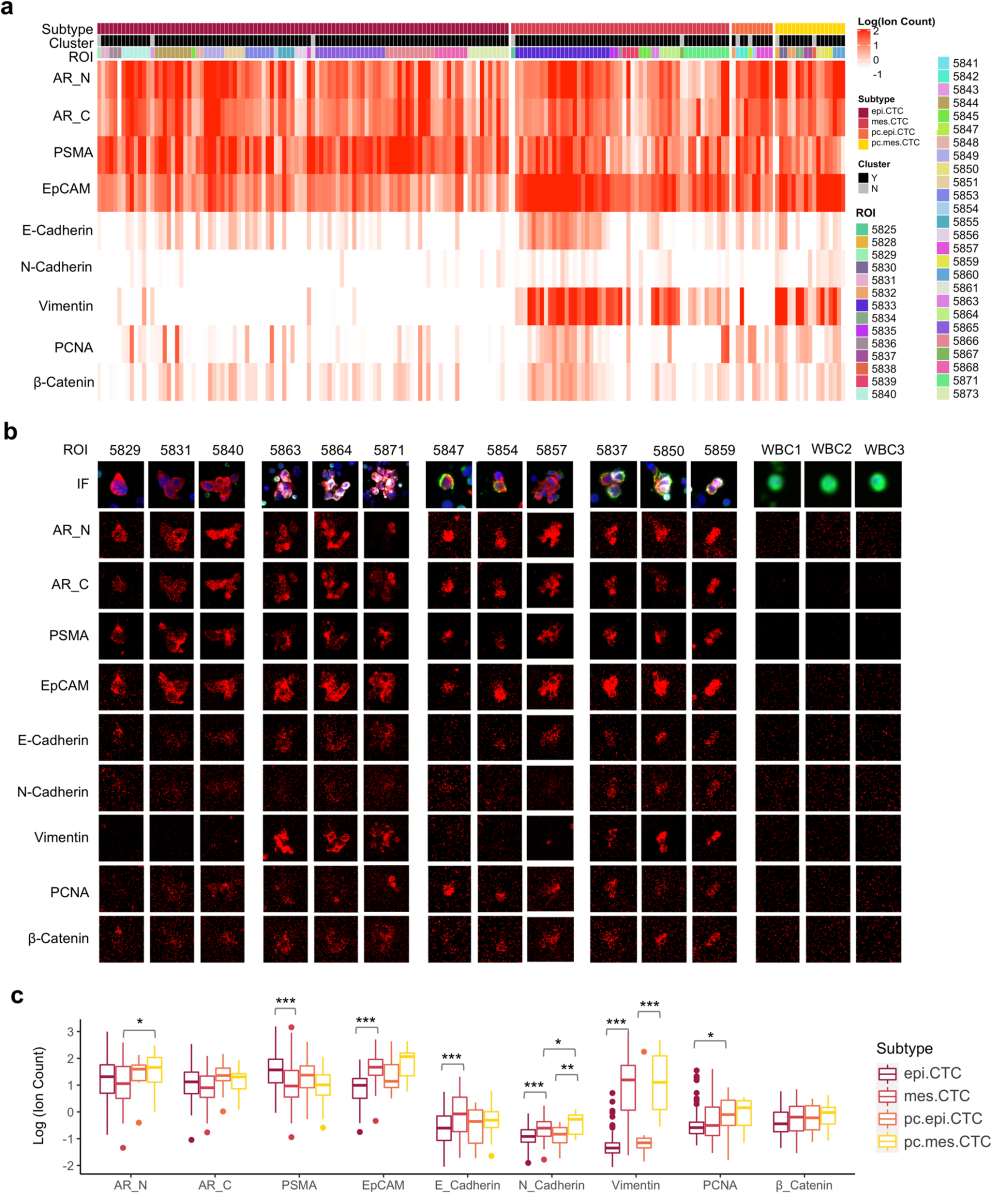
Targeted proteomics of CTC subtypes. **a** Heatmap of proteomic expression in CTC subtypes, including AR-N terminal (AR_N), AR-C terminal (AR_C), PSMA, EpCAM, E-Cadherin, N-Cadherin, Vimentin (VIM), PCNA, and β-catenin. **b** Immunofluorescence and image mass cytometry images of representative CTC subtypes. **c** Comparisons of proteomic expressions among CTC subtypes. Center line of box: median; upper/lower hinges of box: 75% or 25% quartile; upper/lower whiskers of box: hinge ± 1.5*IQR (inter-quartile range). *p < 0.05, **p < 0.01, ***p < 0.001.

The results of targeted proteomic analysis by imaging mass cytometry are presented as an expression heatmap (Fig. 5a) and CTC images from scanned regions of interest (ROI) (Fig. 5b). The combined results show that EpCAM and prostate-specific biomarkers including AR and PSMA were abundantly expressed in all CTC subtypes while negative in white blood cells thus confirming their tissue of origin. VIM expression was significantly higher in mes.CTCs than epi.CTCs, echoing the results of in the immunofluorescence assay (Fig. 5c). PSMA expression was significantly downregulated while EpCAM, E-Cadherin, and N-Cadherin were significantly upregulated in mes.CTCs compared to epi.CTCs, and similar differences were observed between pc.mes.CTCs and pc.epi.CTCs despite not rising to statistical significance (Fig. 5c). In addition, we observed a trend of increasing AR and PCNA in pc.CTCs compared to CTCs (Fig. 5c). Overall, a spectrum of proteomic heterogeneity was observed across and within CTC subtypes (Fig. 5a–b). Besides, all 31 CTC clusters showed various intra-cluster proteomic heterogeneity. Examples include cluster 5871, where AR, PSMA and PCNA were uniquely expressed in top right two cells of cluster and in cluster 5854, the pc.epi.CTC had higher expression of PSMA, EpCAM, PCNA and beta-catenin compared to the adjacent epi.CTC.

## Discussion

Phenotypic heterogeneity of tumor cells along disease progression arises from somatic mutations, adaptation to new microenvironments, or resistance against treatments. The purpose of this study was to distinguish different phenotypic states, particularly epithelial-like and mesenchymal-like states, of CTCs with clonal identity and along clonal lineage to analyze genotype-phenotype association and understand CTC biology in mPC. Previous studies from our group^6,9^ and others^23,24^ have shown the feasibility of single cell multi-omic approach, e.g., immunostaining, whole genome/exome sequencing, transcriptomic profiling, to simultaneously dissect phenotypic and genotypic heterogeneity of detected or in vitro cultured CTCs. HDSCA3.0, as our most upgraded liquid biopsy workflow, utilizes immunofluorescence (DAPI, CK, VIM, CD45/CD31) to characterize mesenchymal feature and platelet attachment status of CTCs followed with either single cell copy number profiling or targeted proteomics. Here, we applied this approach to paired PB and BMA samples from one prostate cancer patient with bone metastasis and fast progression following multiple lines of treatments including hormonal therapies and chemotherapies. In this index patient, we have identified a subgroup of genetically clonal CTCs that acquired a new phenotypic state, i.e., mesenchymal-like state with the additional expression of VIM, which shows the potential of molecular characterization of EMT on genetically confirmed CTCs in different compartments of fluid microenvironment.

Most importantly, the presence of the mesenchymal-like state in CTCs was significantly associated with a genetic subclone emerged along the clonal lineage. Prior studies have revealed that not only epigenetic events and environmental factors could drive EMT trajectories through transcriptional changes, but also genomic alterations could impact this cell state transition^25^. For example, *CAMK1D* (localized at chr.10p13) was highly expressed in basal-like breast cancer due to its gene amplification and exogeneous overexpression could induce EMT^26^. Similarly, high expression of *PLS3* was significantly related to copy number gain of chr.Xq23 which is its genetic locus and promoted EMT through transforming growth factor (TGF)-β signaling in colorectal cancer cells^27,28^. Oppositely, *SLC38A3* (localized at chr.3p21) expression was lower in the tumor tissue than the adjacent normal tissue, linked to its high frequency of gene deletion and further experiments revealed that deletion of *SLC38A3* could stimulate EMT in esophageal squamous cell carcinoma^29^. However, those potential associations were inferred from the observations in bulk tumor samples or the validations in cell lines, while intra-patient genotype-phenotype relationship at single cell level is still unknown, especially in CTCs. Here, our clonal lineage tracing of CTCs has shown that there were at least 3 distinctive subclones emerged under the main clone, include clade2, clade3, and clade4/5, based on the optimal number of clades from hierarchical clustering determined in a mathematical way. More interestingly, the VIM + group had significantly higher percentage of the clade4/5 subclone while the VIM- group was enriched with the clade2 subclone. Further comparison analysis between those two main subclones (clade2 vs clade4/5) had demonstrated a variety of different chromosome or gene level CNAs across the whole genome. We hypothesized the reasons to be that genetic defects of EMT-related genes could cause mesenchymal transformation in clade4/5 subclone or loss of cell state transition ability in clade2 subclone, e.g., copy number gains of *PDGFRA, PIK3CA*, etc in the clade4/5 subclone while copy number loss of *TWIST2, JAK2, etc* in the clade2 subclone. In addition to 4 subtypes of CTCs, we also included “VIM + only” classification of cells to represent the full spectrum of rare cells detected and to further investigate the potential of CTCs that completed the EMT and lost epithelial biomarker, e.g., cytokeratin^30,31^. Single cell copy number profiling showed those cells did not carry the cancer cell genomic architecture (the main clone) which is consistent with our observation in the previous publication^9^, demonstrating clonality in CK + cell groups in metastatic prostate cancer (mPC). Meanwhile, recent studies have shown hybrid and dynamic phenotypes generated from EMT of CTCs which enhance adaptation ability, treatment resistance, and metastatic potential^25,32,33^. We acknowledge that the mesenchymal phenotype characterized in our immunostaining has yet to be sufficient to depict the heterogeneity of EMT. We are developing new IF-based assays which will include additional biomarkers to further characterize the plasticity of EMT on CTCs, as well as transcriptomic profiling using single cell RNA sequencing.

Spatial heterogeneity of CTCs during metastasis across multiple liquid compartments has been reported at the transcriptomic level perhaps as an adaptation to different circulation microenvironments^34^. Here, we investigated the phenotypic and genotypic differences of CTCs from two different compartments of the fluid microenvironment during tumor metastasis. Consistent with phenotypic differences in our previous studies^6,21^, the abundance and phenotypic diversity of CTCs in the BMA are significantly higher than in the PB which indicates site-specific enrichment and enhanced heterogeneity of CTCs adapting to the bone marrow microenvironment. In terms of genotypic differences, the clade4/5 was uniquely detected in the 66.2% (45/68) of clonal CTCs from BMA as the dominant subclone, while the clade1 minor clone with chromosome 13 and 22 losses was only detected in PB. Interestingly, the cells in the minor clone are all VIM positive with or without CK expression, likely to be circulating TME cells with clonal alterations while the presence of RB1 and BRCA2 losses in the minor clone indicated the probability of the generation of a new cancer clone. To determine if they are TME cells with clonal alteration or CTCs with the new cancer clone, additional biological characterization is warranted. Due to high CTC abundance and additional observation of phenotypic and genotypic heterogeneity in BMA, despite its invasiveness, we suggest that BMA could be a robust supplemental approach, especially when CTC is not detected in PB. Meanwhile, BMA is still less invasive than prostate biopsy and also provides a unique perspective of metastatic cancer cells which usually present aggressive behavior.

CTC cluster or aggregate is another unique phenotype and studies has illustrated its role of indicating worse prognosis^21^ as well as the epigenetic regulations during its formation^35^. Here, we explored the genotypic and phenotypic heterogeneity of CTCs from the same clusters. Out of 14 CTC clusters from the BMA, 4 of them had heterogeneous subclones and the rest of them possessed the homogeneous subclones. This observation relates to the hypothesis of CTC cluster formation: (1) the cluster with the same subclone could be formed by cell division from one parental cell or cell aggregation of tumor cells with the same genetic background; (2) the cluster with different subclones could be formed by physical aggregation between 2 or more cells with various genetic backgrounds. It will be interesting to investigate different mechanisms of CTC cluster formation as well as their potential clinical significance.

Overall, this case report demonstrated a robust single cell multi-omic approach to simultaneously deconvolute genotype and phenotype of CTCs in paired liquid biopsy samples from bone mPCs. This approach allows for a complete spatial evolution analysis of cancer metastasis and provides the opportunity to compare multiple compartments of liquid biopsies. The uniqueness of this case includes the confirmation of mes.CTCs (CK + VIM + ) with cancer cell identity, the observation of clonal heterogeneity across subtypes and samples, and, most importantly, morpho-genomic analysis connects a subclonal genotype with an EMT phenotype at the single cell level and elucidates biological heterogeneity and complexity of CTCs.

## Methods

### Patient selection and sample preparation

The index patient in this study was diagnosed with de novo mPC and following castrate resistance and further disease progression, paired PB and BMA samples were collected and shipped to University of Southern California within 24 hours of the collection for analysis with the HDSCA3.0 workflow^9^. This patient was registered under the pre-enrollment of “*cabazitaxel with or without carboplatin*” trial (NCT01505868). The study was approved by the Institutional Review Board and adhered to the principles in the Declaration of Helsinki. The patient provided written informed consent prior to inclusion in the study.

Sample preparation was performed as previously described^5,6,21,36^. Briefly, upon arrival in the laboratory, both PB and BMA liquid biopsy samples were treated with isotonic ammonium chloride solution for erythrocyte removal and the isolated nucleated cells from centrifuging were plated on the cell-adhesive slides (Marienfeld) and stored at −80 °C.

### Immunofluorescent staining and scanning

Two slides with ~3 million nucleated cells each from PB and BMA, respectively, were stained according to the HDSCA3.0 protocol as previously described^9^. Briefly, slides were thawed for 1 h, fixed with 2% formalin for 20 min, and incubated with 10% goat serum for another 20 min. The following staining steps were conducted on an IntelliPATH FLX autostainer (Biocare Medical LLC) with negative and positive control slides included: (1) a mixture containing an anti-human CD31:Alexa Fluor 647 mouse IgG1 monoclonal Antibody (BioRad; Cat# MCA1738A647; Clone: WM59; Working concentration: 2.5 μg/mL) and an anti-mouse IgG goat monoclonal Fab fragment (Jackson ImmunoResearch; Cat# 115–007–003; Working concentration: 100 μg/mL) for 4 h; (2) 100% cold methanol for permeabilization for 5 min; (3) a mixture consisting of an anti-human cytokeratin (CK) 1,4,5,6,8,10,13,18,19 mouse IgG1/IgG2a monoclonal antibody cocktail (Sigma; Cat# C2562; Clone: C-11, PCK-26, CY-90, KS-1A3, M20, A53-B/A2; Working concentration: 210 μg/mL), an anti-human CK 19 mouse IgG1 monoclonal antibody (Dako; Cat# GA61561–2; Clone: RCK108; Working concentration: 0.2 μg/mL), an anti-human CD45:Alexa Fluor 647 mouse IgG2a monoclonal antibody (AbD Serotec; Cat# MCA87A647; Clone: F10–89–4; Working concentration: 1.2 μg/mL), and an anti-human vimentin (VIM): Alexa Fluor 488 rabbit IgG monoclonal antibody (Cell Signaling Technology; Cat# 9854BC; Clone: D21H; Working concentration: 3.5 μg/mL) for 2 h; (4) a mixture including an anti-mouse IgG1: Alexa Fluor 555 goat IgG polyclonal antibody (Invitrogen; Cat# A21127; Working concentration: 2 μg/mL) and 40,6-diamidino-2-phenylindole (DAPI) for nuclear DNA (Thermo Fisher Scientific; Cat# D1306; Dilution: 1: 50,000) for 1 h. Finally, slides were mounted with a glycerol-based aqueous mounting media followed with adding coverslips. Slides were then scanned, as previously described^9^. Briefly, slides were scanned using an automated fluorescence scanning microscopy at ×10 objective magnification and generating 2304 frame images for each channel (DAPI: DNA; Alexa Fluor 555: CK; Alexa Fluor 488: VIM; Alexa Fluor 647: CD45/CD31). Exposure time and gain were automatically set up to yield the same background intensity level across slides before auto-scanning. Some cells on the slides were further manually re-imaged using a fluorescence microscopy at ×40 objective magnification for higher resolution images.

### Rare cell detection and CTC subtype enumeration

Image analysis was performed as previously reported^9^. In brief, the EBImage package^37^ was used to segment DAPI + (cell) events by generating nuclear and/or cytoplasm masks. For each segmented event, 761 quantitative cellular and nuclear features were extracted by “computeFeatures” in the EBImage package and top 350 principal components were further identified from extracted features by principal component analysis. Hierarchical clustering was performed among detected cells with those principal components of features to separate common cells (mainly leukocytes) and rare cells in each frame image.

Manual classification of rare cells into CTC subgroups was also previously reported^9^. Briefly, epi.CTCs were classified as cells are CK positive, VIM negative and CD45/CD31 negative with distinctive nucleus morphology and mes.CTCs gained VIM expression in addition to CK. Platelet attachment, featured as punctuated CD45/CD31 signals on cell surface, further categorized platelet-coated (pc) epi.CTCs (pc.epi.CTCs) and mes.CTCs (pc.mes.CTCs).

### Single cell copy number profiling by low pass whole genome sequencing

Single cell copy number profiling was performed as previously reported^5,6,9,21^. Briefly, cells of interest were relocated using XY coordinates generated from the scanning and 40× high-quality images were captured before the single cell isolation by micromanipulator. Individual single cells were lysed for whole genome amplification (Sigma-Aldrich; Cat# WGA4) and libraries were constructed using the DNA Ultra Library Prep Kit (New England Biolabs; Cat# E7370) and sequenced by Illumina NextSeq 500 at USC for single-end 50 bp read sequencing. Following genome mapping and PCR duplicate removal, unique reads were seated into ~5000 pre-defined bins and the number of reads per bin was normalized as “ratio to mean” for constructing copy number profile and heatmap and identifying copy number alteration (CNA). The dendrogram on the heatmap was grouped by hierarchical clustering using “cluster-agnes” R package (metric = Manhattan, method = ward). Large scale transition (LST) was a measurement of the number of large scale (>10 Mb) CNAs across the whole genome^38^. Minimum evolution method^39^ was used for phylogenetic tree construction of individual cells or clade consensus (the median of cells from the same clade). The contamination of normal cell in CTC could compress the copy number profiles and was manually identified and labeled as “c” standing for “compressed”. The evaluation was based on checking if the “ratio to mean” of CNA was matched with the theoretical ratio between the integer copy number of gain/loss region and the ploidy number of one single cell, e.g., in a diploid cell, the theoretical ratio for one copy loss is 0.5 (1/2) and the value for one copy gain is 1.5 (3/2). In terms of the cell selection criteria for single cell genomics, we sequenced cells across different subtypes including epi.CTC, mes.CTC, pc.epi.CTC, and pc.mes.CTC to observe genotypic heterogeneity and aimed for at least five cells analyzed per subtype per sample in consideration of the number of cells detected and the number of cells successfully isolated and sequenced.

### Targeted proteomics by imaging mass cytometry

Cells of interest were subjected to in situ targeted proteomic analyses with the use of the CyTOF Helios imaging mass cytometer (Fluidigm) as previously described^6,40^. Briefly, sample slides were re-stained with metal-conjugated antibodies (Prostate-specific: AR-N (Cell Signal Technology; Cat# 5153; Clone: D6F11; Dilution: 1:200), AR-C (LS Bio; Cat# LS-C210456-500; Clone: SP242; Dilution: 1:200), PSMA (Novus; Cat# MAB4234; Clone: 460420; Dilution: 1:200); EMT: EpCAM (Fluidigm; Cat# 3144026D; Clone: 9C4; Dilution: 1:200), E-cadherin (Fluidigm; Cat# 3158029D; Clone: 24E10; Dilution: 1:300), Vimentin (Abcam; Cat# ab193555; Clone: EPR3776; Dilution: 1:300), N-cadherin (Abcam; Cat# ab19348; Clone: 8C11; Dilution: 1:200); Cell-proliferation: PCNA (Abcam; Cat# ab18197; Clone: Polyclonal; Dilution: 1:400), β-catenin (Fluidigm; Cat# 3147005 A; Clone: D10A8; Dilution: 1:300) and a DNA intercalator. Antibodies that were not available with Fludigm were sourced from the third-party vendors and were custom conjugated in the lab. Maxpar antibody labeling kits were used to label the antibodies with the metals of choice. Metal labelled antibodies cocktail was applied to the experiment slide during staining process. A region of interest (ROI) of ~400 µm × 400 µm centered on each candidate cell was ablated with a 1 µm diameter pulsed laser, followed by ionization and quantification in the CyTOF Helios instrument. Ion mass data were collected and used for reconstruction of the 1 µm^2^ ROI spatial resolution, multi-dimensional images of the ROI. Cell segmentation and ion count per cell were generated by IMC segmentation pipeline created by the Bodenmiller Lab^41^, based on the CellProfiler (version 3.15)^42^ and Ilastik (version 1.3.3)^43^ and images with segmented masks could be further displayed by HistoCAT^44^.

### R packages and statistical analysis

For data visualization, we used t-SNE (version 0.15)^45^ for dimensionality reduction, ggplot2 (version 2.8.0)^46^ for scatter or bar plots, ape (version 5.5)^47^ and ggtree (version3.0.4)^48^ for phylogenetic tree, and Complex Heatmap (version 3.3.5)^49^ for heatmaps. Chi-square was used for categorical data association analysis and Mann–Whitney U test was used for non-parametric data (i.e., ion count).

### Reporting summary

Further information on research design is available in the Nature Research Reporting Summary linked to this article.

## Supplementary information

Supplementary Information

REPORTING SUMMARY

## Data Availability

All data discussed in this paper are either included in the main figures or the supplementary files. The single cell sequencing data is available through the Sequence Read Archive with BioProject accession number PRJNA827940. The immunofluorescence image data is available in figshare at 10.6084/m9.figshare.19617717.v1. The image mass cytometry data is available in figshare at 10.6084/m9.figshare.19619007.v1.

## Code availability

The code used to analyze the single cell sequencing and image mass cytometry data uses standard third-party open-source libraries and packages in R and/or Python. The code used to identify CTCs in the HDSCA3.0 workflow uses custom code and is proprietary and licensed to Epic Sciences for commercial use.
