# Supplementary material for: Identification of epithelial and mesenchymal circulating tumor cells in clonal lineage of an aggressive prostate cancer case
Source: NPJ Precis Oncol. 2022 Jun 21;6:41. doi: 10.1038/s41698-022-00289-1 (PMC9213535; doi:10.1038/s41698-022-00289-1)
Supplement: Supplementary file 1 — Supplementary Information [file 41698_2022_289_MOESM1_ESM.pdf]

Supplementary Information

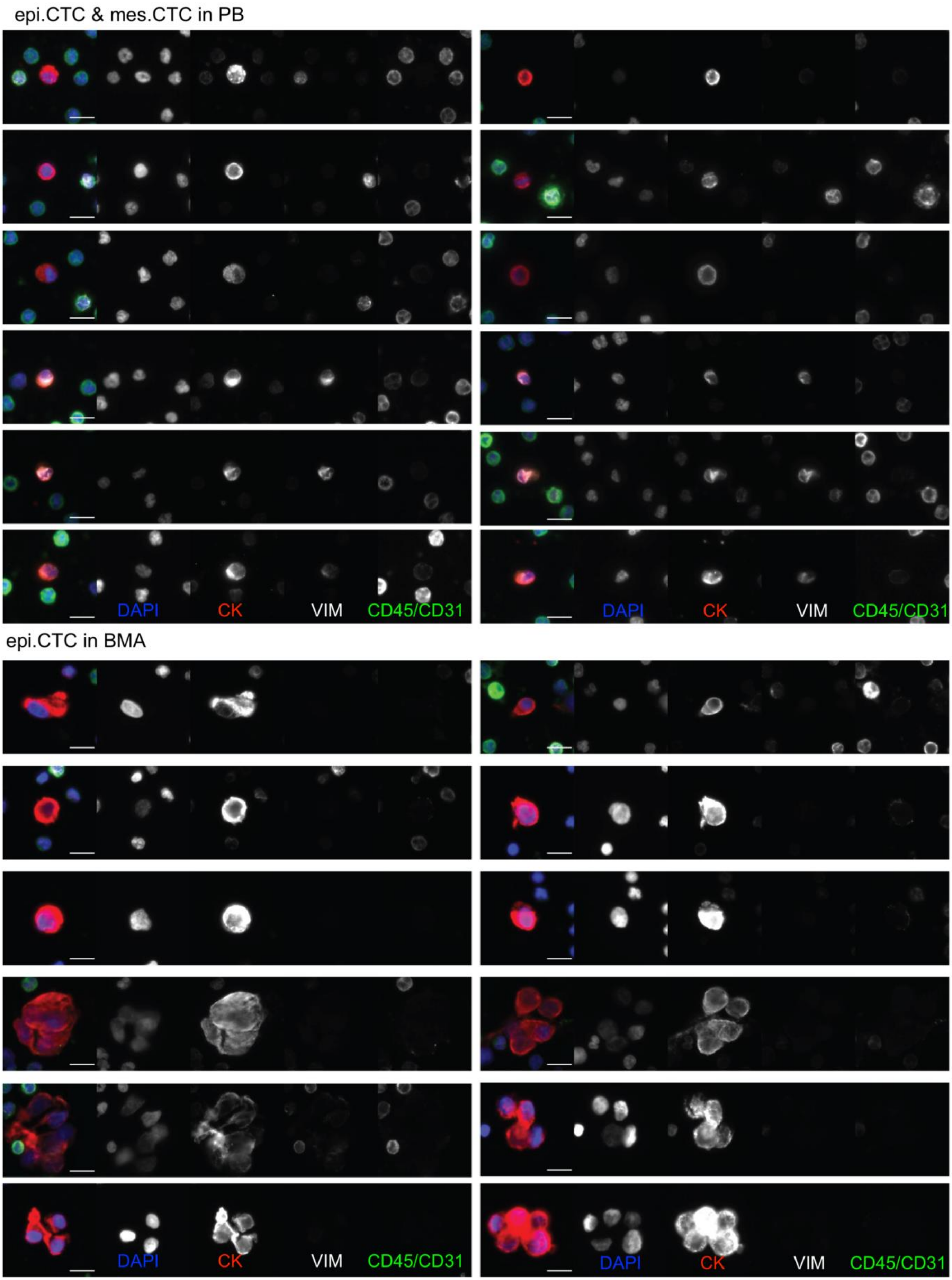

mes.CTC in BMA

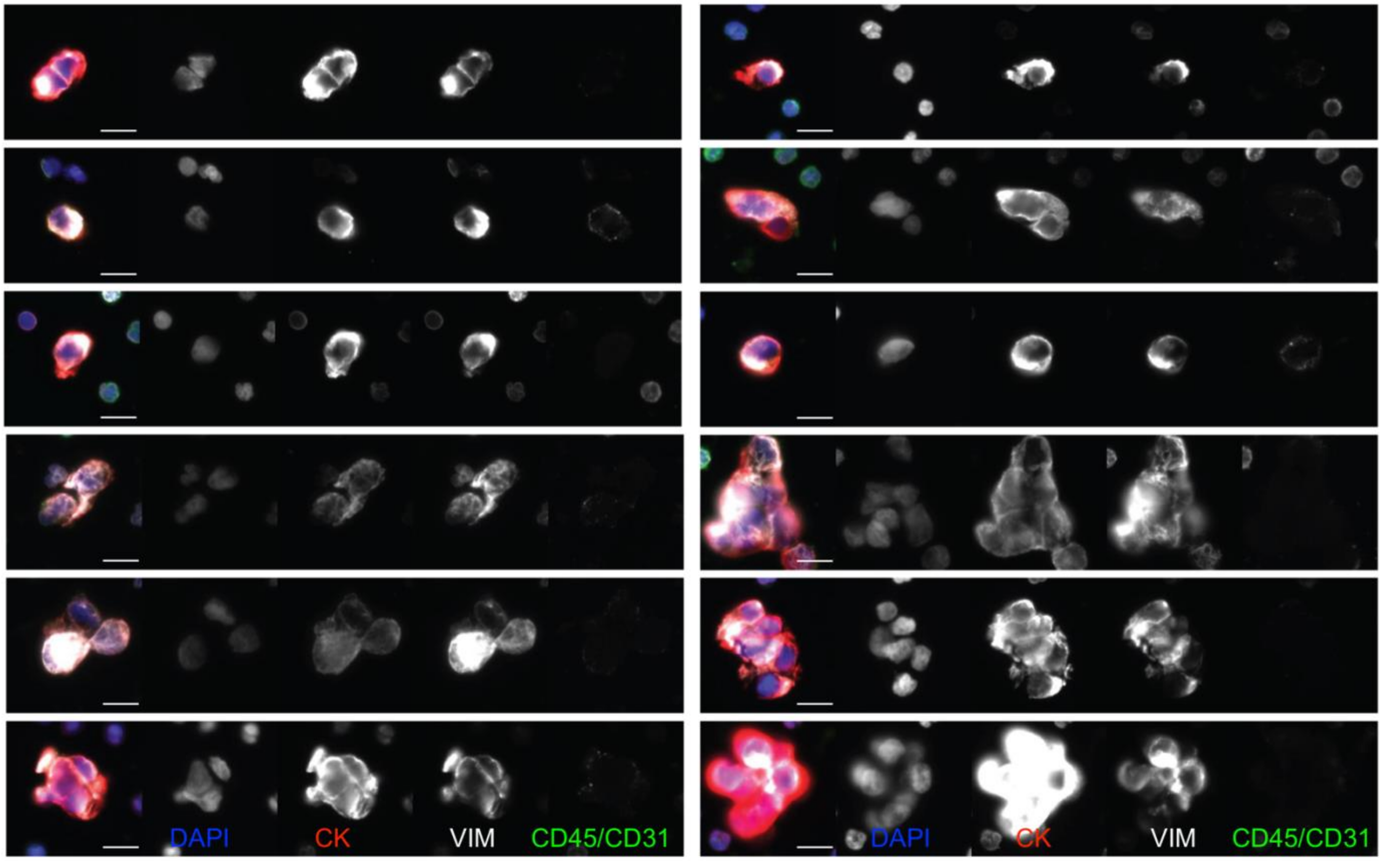

pc.epi.CTC & pc.mes.CTC in BMA

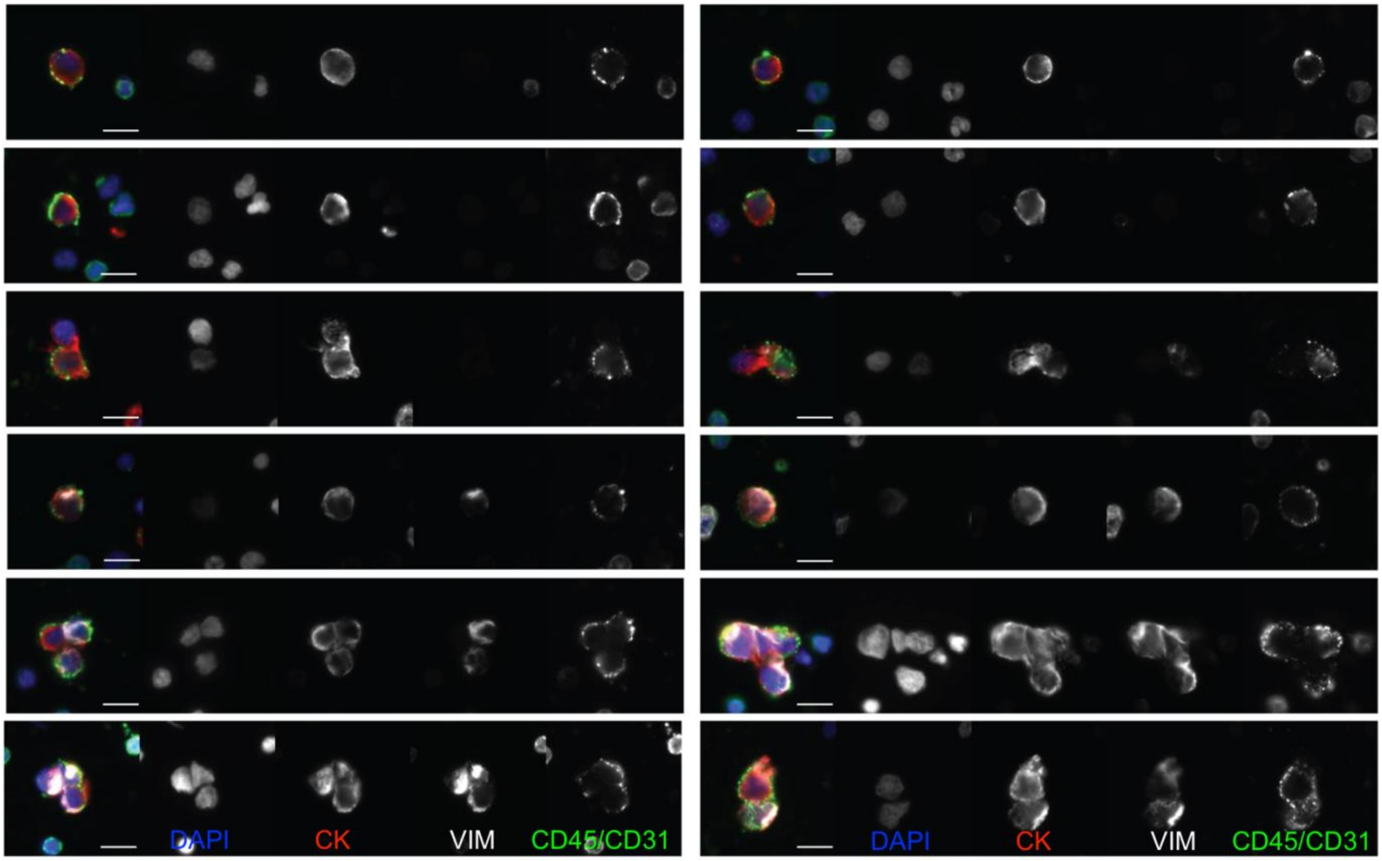

Supplementary Figure 1. Immunofluorescence composite and all-channel images of CTCs from PB sample and BMA sample. Color coding: DAPI (blue); CK (red); VIM (white); CD45/CD31 (green). Scale bar: 10μm.

a

Nonrelevant features removal → Spearman correlation analysis → Select representative features from individual highly correlated groups →

1. mean intensity of DAPI channel (nucleusf.dapi.b.mean)
2. mean intensity of TRITC channel (cellf.tritc.b.mean)
3. mean intensity of FITC channel (cellf.fitc.b.mean)
4. mean intensity of CY5 channel (cellf.cy5.b.mean)
5. cell radius (cellf.0.s.radius.mean)
6. nucleus radius (nucleusf.0.s.radius.mean)
7. cell eccentricity (cellf.0.m.eccentricity)
8. nucleus eccentricity (nucleusf.0.m.eccentricity)
9. cell-nucleus ratio of sizes (cell_nucleus_ratio)
10. Nucleus location in cell (nucleus_cell_dist)

b

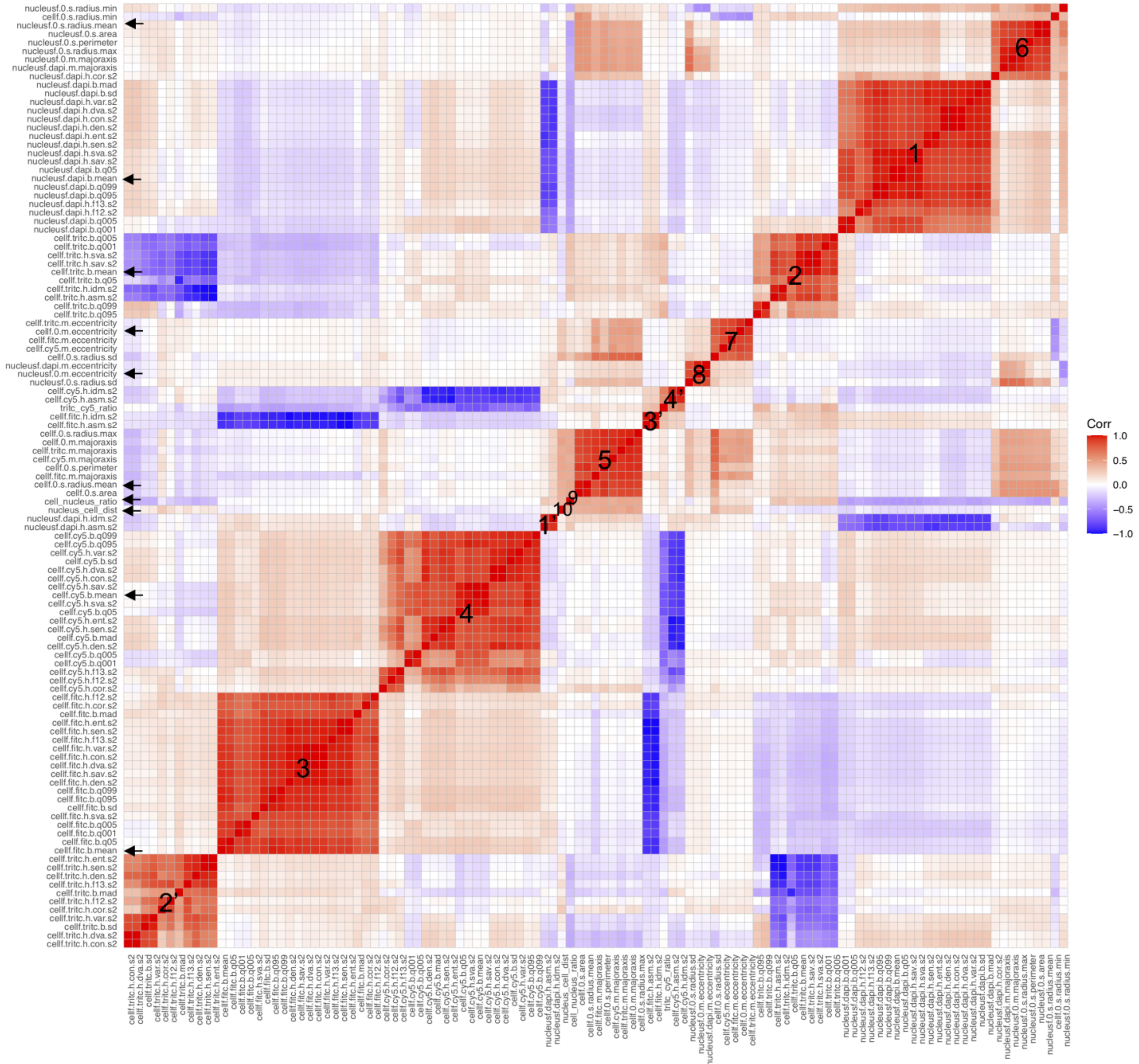

Supplementary Figure 2. Feature selection pipeline and correlation analysis. a. Image features selection pipeline including 1) non-relevant features removal – 2) Pearson correlation analysis – 3) Representative feature selection within each group – 4) 10 selected features. **b**. Ten groups of highly correlated features identified through Pearson correlation analysis. 1', 2', 3', 4' are negatively correlated features with features in group 1, 2, 3, 4. Arrows are the selected features for each group.

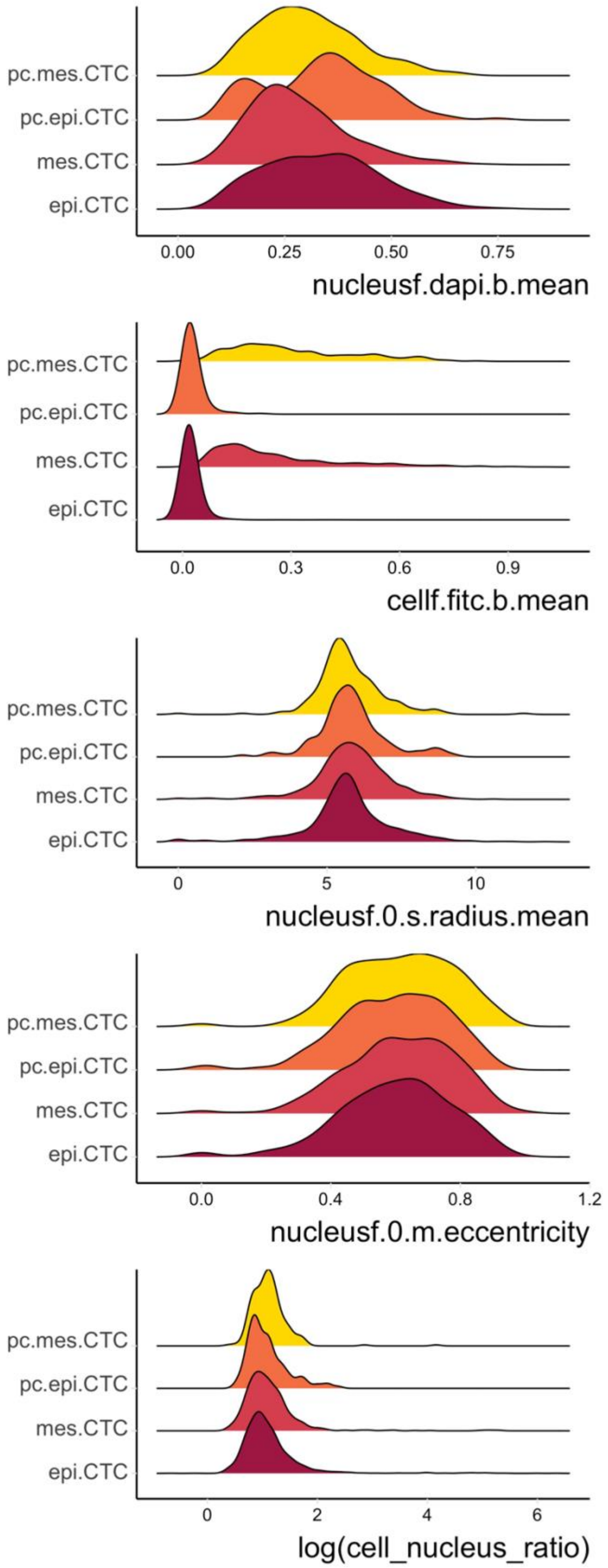

Supplementary Figure 3. Intensity and morphological features comparison across CTC subtypes.

a

	clade2	clade4	clade5	Genes
+2p	N	N	Y	MYCN, NCOA1, DNMT3A, ALK, EML4, MSH2, FBXO11
-2q	Y	N	N	ERCC3, CXCR4, ZEB2
+3p	N	Y	Y	SRGAP3, FANCD2, PPARG, RAF1
+3q	N	Y	Y	GATA2, FOXL2, MLF1, PIK3CA, SOX2, ETV5, EIF4A2, BCL6
+4q	N	Y	Y	KIT, PDGFRA, MLLT2, TET2
-4q	Y	N	N	ACSL1, CASP3, TLR3
+6q	N	Y	Y	ROS1, MYB, TNFAIP3, ECT2L, FGFR1OP, MLLT4
-9p	Y	N	N	JAK2, CD273, NFIB, MLLT3, CDKN2A, FANCG, PD-L2
+9q	N	Y	Y	PAX5, SYK5, FANCC, NR4A3, TAL2, KLF4, NOTCH1
+11p	Y	N	N	FANCF, PIK3C2A
+13q	N	Y	Y	CDX2
-14p	Y	N	N	FOXA1, RAD51L1
+14q	Y	N	N	TSHR, TRIP11, HSPCA
+19p	N	Y	Y	MLLT1, JAK3, ELL
+21q	Y	N	N	RUNX1, ERG
+Xp1	Y	N	N	CRLF2, P2RY8, ZRSR2
+Xp2	N	Y	Y	GATA1, KDM5C

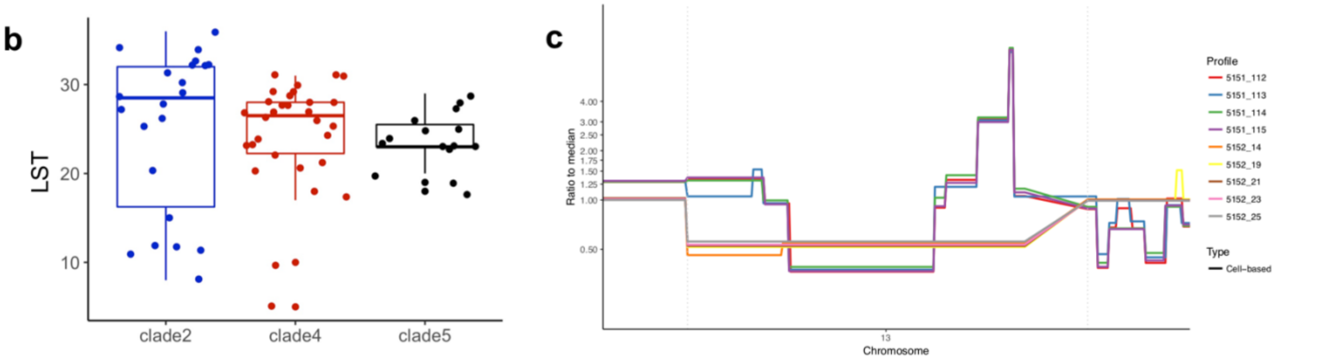

Supplementary Figure 4. Genomic comparisons among subclones. a. Chromosomal and gene copy number alteration differences among genomic subclones. Y: positive for CNA; N: negative for

CNA. **b**. Comparison of LST among genomic subclones. **c**. Breakpoint difference of copy number alterations on chromosome 13 between main clone cells and minor clone cells.

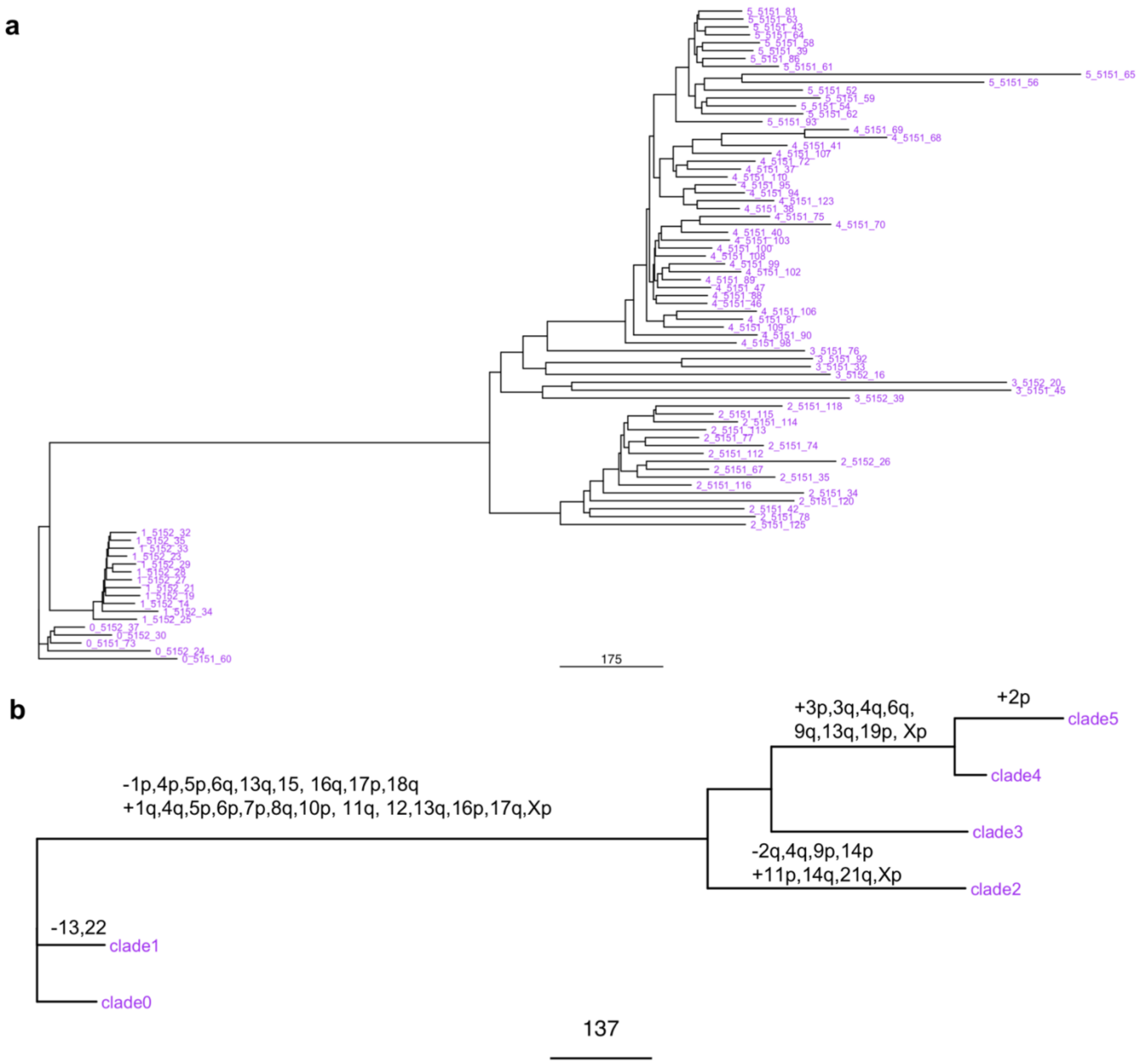

Supplementary Figure 5. Single cell and clonal lineage trees. a. Minimum evolution tree of single cell copy number profiles (cells with diluted profiles were excluded) with Manhattan distance. The first number is the clade ID, the second number is the sample ID (BMA: 5151; PB: 5152), and the third number is the sequencing cell ID. **b**. Minimum evolution tree of median copy number profiles of clades with Manhattan distance.

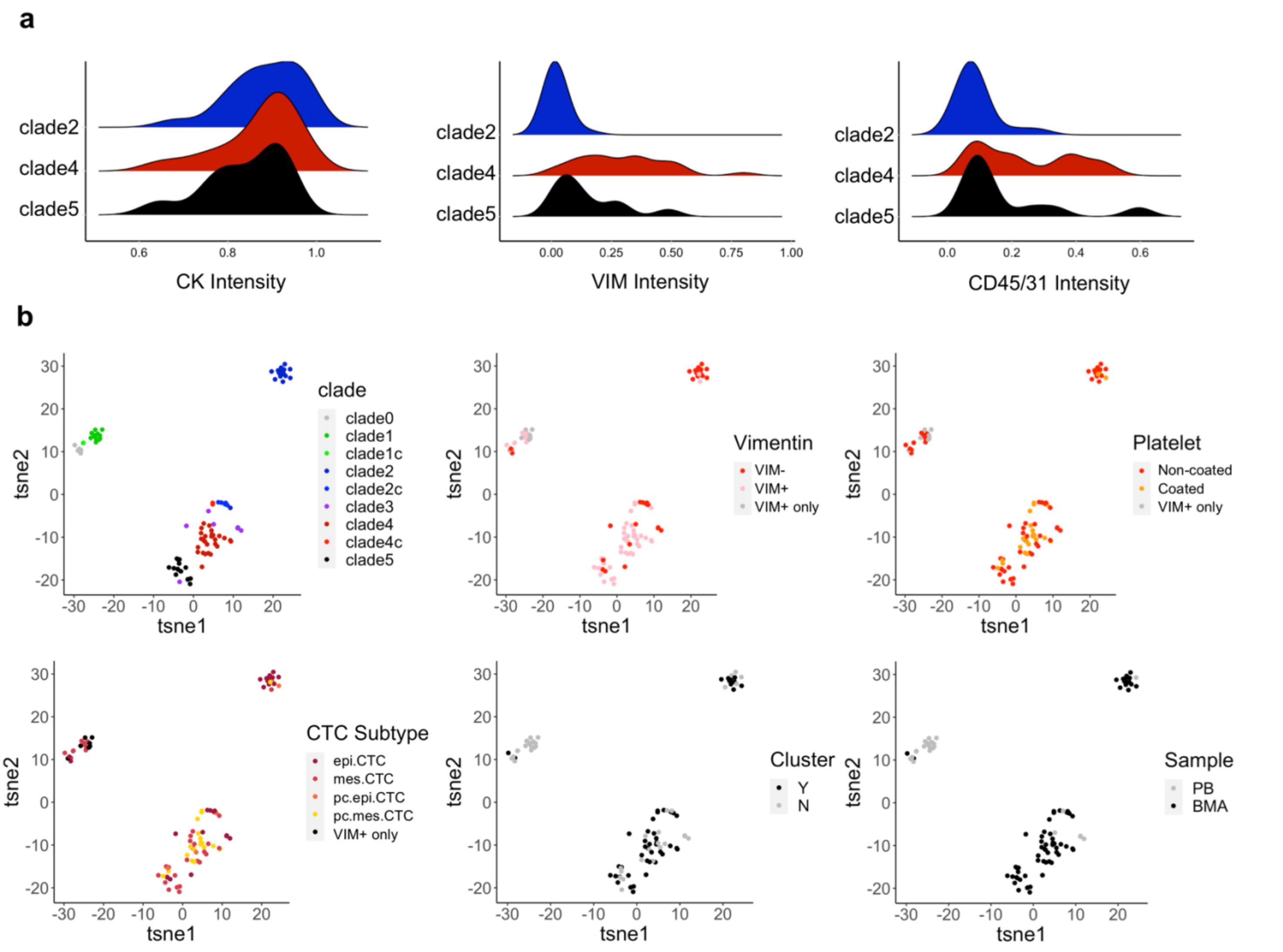

Supplementary Figure 6. Association between genomic subclones and phenotypes. a. Fluorescence intensity distributions in three different subclone groups. **b**. t-SNE analysis for dimensionality reduction of copy number ratios of 5k bins labeled with hierarchical clustering based subclones, morpho-proteomic features and sample types

a

Cluster	Cell ID	clade0	clade2	clade3	clade4	clade5	Total	Hetero
Cluster1	54-59					4	4	N
Cluster2	60-65	1				5	6	N
Cluster3	67-68		1		1		2	Y
Cluster4	69-70				2		2	N
Cluster5	72-75	1	1		2		4	Y
Cluster6	76-77		1	1			2	Y
Cluster7	92-93			1		1	2	Y
Cluster8	94-95				2		2	N
Cluster9	99-103				4		4	N
Cluster10	105-106				2		2	N
Cluster11	107-110				4		4	N
Cluster12	111-115		5				5	N
Cluster13	116-117		2				2	N
Cluster14	124-125		2				2	N

b

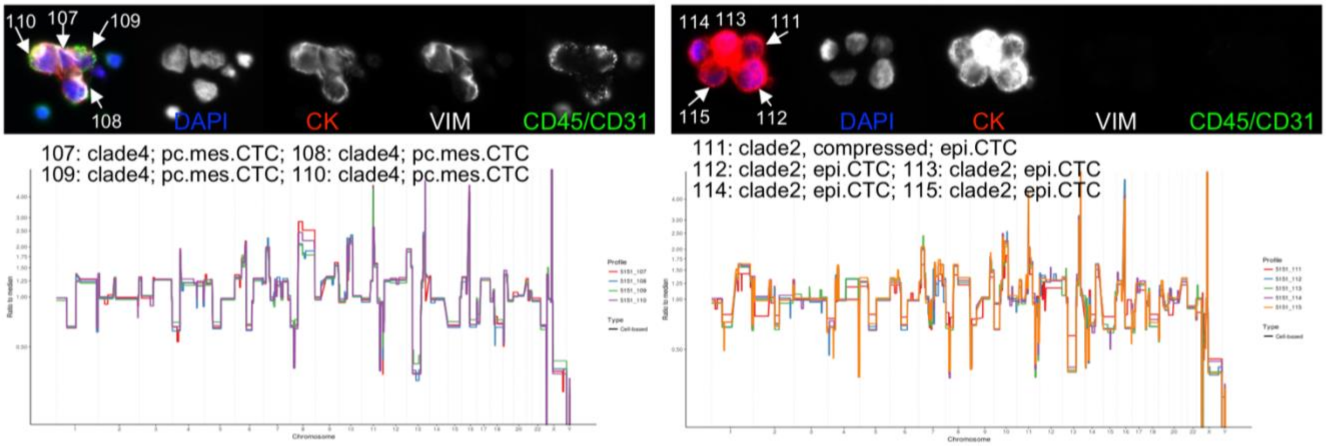

Supplementary Figure 7. Intra-cluster characterization of genomic heterogeneity. **a**. Table represents the clonality of single cells from 14 CTC clusters in the BMA sample. Y: heterozygous; N: homozygous. The cell ID is the sequencing cell ID. **b**. Immunofluorescence images and single cell copy number profiles of representative homogeneous CTC clusters. Images order: composite, DAPI, CK, VIM, and CD45/CD31.
