# Supplementary material for: Identification of epithelial and mesenchymal circulating tumor cells in clonal lineage of an aggressive prostate cancer case
Source: NPJ Precis Oncol. 2022 Jun 21;6:41. doi: 10.1038/s41698-022-00289-1 (PMC9213535; doi:10.1038/s41698-022-00289-1)
Supplement: Supplementary file 2 — REPORTING SUMMARY [file 41698_2022_289_MOESM2_ESM.pdf]

nature portfolio

Corresponding author(s): Peter Kuhn, PhD; James Hicks, PhD

Last updated by author(s): Feb 16, 2022

Reporting Summary

Nature Portfolio wishes to improve the reproducibility of the work that we publish. This form provides structure for consistency and transparency in reporting. For further information on Nature Portfolio policies, see our Editorial Policies and the Editorial Policy Checklist.

Statistics

For all statistical analyses, confirm that the following items are present in the figure legend, table legend, main text, or Methods section.

n/a	Confirmed	
☐	☒	The exact sample size (*n*) for each experimental group/condition, given as a discrete number and unit of measurement
☐	☒	A statement on whether measurements were taken from distinct samples or whether the same sample was measured repeatedly
☐	☒	The statistical test(s) used AND whether they are one- or two-sided<br>*Only common tests should be described solely by name; describe more complex techniques in the Methods section.*
☒	☐	A description of all covariates tested
☒	☐	A description of any assumptions or corrections, such as tests of normality and adjustment for multiple comparisons
☐	☒	A full description of the statistical parameters including central tendency (e.g. means) or other basic estimates (e.g. regression coefficient) AND variation (e.g. standard deviation) or associated estimates of uncertainty (e.g. confidence intervals)
☐	☒	For null hypothesis testing, the test statistic (e.g. *F*, *t*, *r*) with confidence intervals, effect sizes, degrees of freedom and *P* value noted<br>*Give P values as exact values whenever suitable.*
☒	☐	For Bayesian analysis, information on the choice of priors and Markov chain Monte Carlo settings
☒	☐	For hierarchical and complex designs, identification of the appropriate level for tests and full reporting of outcomes
☒	☐	Estimates of effect sizes (e.g. Cohen's *d*, Pearson's *r*), indicating how they were calculated

Our web collection on statistics for biologists contains articles on many of the points above.

Software and code

Policy information about availability of computer code

Data collection	The code used to analyze the single cell genomics and targeted proteomics data uses standard third-party open-source libraries and packages in R and/or Python. The code used to identify CTCs in the HDSCA3.0 workflow uses custom code and is proprietary and licensed to Epic Sciences for commercial use.
Data analysis	For data visualization, we used t-SNE (version 0.15) for dimensionality reduction, ggplot2 (version 2.8.0) for scatter or bar plots, ape (version 5.5) and ggtree (version3.0.4) for phylogenetic tree, and Complex Heatmap (version 3.3.5) for heatmaps. Chi-square was used for categorical data association analysis and Mann-Whitney U test was used for non-parametric data (i.e., ion count).

For manuscripts utilizing custom algorithms or software that are central to the research but not yet described in published literature, software must be made available to editors and reviewers. We strongly encourage code deposition in a community repository (e.g. GitHub). See the Nature Portfolio guidelines for submitting code & software for further information.

Data

Policy information about availability of data

All manuscripts must include a data availability statement. This statement should provide the following information, where applicable:

- Accession codes, unique identifiers, or web links for publicly available datasets
- A description of any restrictions on data availability
- For clinical datasets or third party data, please ensure that the statement adheres to our policy

All data discussed in this manuscript are either included in the main figures or the supplementary files. The imaging data, single cell sequencing data, and image mass cytometry data is available through the BloodPAC Data Commons Accession ID “BPDC000122”. https://data.bloodpac.org/discovery/BPDC000122/

Field-specific reporting

Please select the one below that is the best fit for your research. If you are not sure, read the appropriate sections before making your selection.

☒ Life sciences ☐ Behavioural & social sciences ☐ Ecological, evolutionary & environmental sciences

For a reference copy of the document with all sections, see nature.com/documents/nr-reporting-summary-flat.pdf

Life sciences study design

All studies must disclose on these points even when the disclosure is negative.

Sample size	One peripheral blood and one bone marrow aspirate samples from single patient with metastatic castrate-resistant prostate cancer
Data exclusions	No data was excluded.
Replication	The peripheral blood and one bone marrow aspirate sample can not be reproduced because they were patient-derived samples.
Randomization	This is not relevant to our study. This is a case report with one patient who was in the pre-enrollment of a trial, but failed to participate.
Blinding	This is not relevant to our study. This is a case report with one patient who was in the pre-enrollment of a trial, but failed to participate.

Reporting for specific materials, systems and methods

We require information from authors about some types of materials, experimental systems and methods used in many studies. Here, indicate whether each material, system or method listed is relevant to your study. If you are not sure if a list item applies to your research, read the appropriate section before selecting a response.

Materials & experimental systems

n/a	Involved in the study	
☐	☒	Antibodies
☒	☐	Eukaryotic cell lines
☒	☐	Palaeontology and archaeology
☒	☐	Animals and other organisms
☐	☒	Human research participants
☐	☒	Clinical data
☒	☐	Dual use research of concern

Methods

n/a	Involved in the study	
☒	☐	ChIP-seq
☒	☐	Flow cytometry
☒	☐	MRI-based neuroimaging

Antibodies

Antibodies used	Immunostaining: Pan-Cytokeratin cocktail (pan-CK: Sigma; Cat# C2562; Clone: C-11, PCK-26, CY-90, KS-1A3, M20, A53-B/A2 and CK19: Dako; Cat# GA61561–2; Clone: RCK108), Vimentin (VIM: Cell Signaling Technology; Cat# 9854BC; Clone: D21H3), and a combined cocktail of CD45 (AbD Serotec; Cat# MCA87A647; Clone: F10–89–4) and CD31 (BioRad; Cat# MCA1738A647; Clone: WM59)<br>Image Mass Cytometry: Prostate-specific: AR-N (Cell Signal Technology; Cat# 5153; Clone: D6F11), AR-C (LS Bio; Cat# LS-C210456-500; Clone: SP242), PSMA (Novus; Cat# MAB4234; Clone: 460420); EMT: EpCAM (Fluidigm; Cat# 3144026D; Clone: 9C4), E-cadherin (Fluidigm; Cat# 3158029D; Clone: 24E10), Vimentin (Abcam; Cat# ab193555; Clone: EPR3776), N-cadherin (Abcam; Cat# ab19348; Clone: 8C11); Cell-proliferation: PCNA (Abcam; Cat# ab18197; Clone: Polyclonal), β-catenin (Fluidigm; Cat# 3147005A; Clone: D10A8)
Validation	The performance validation of antibody was described in previous publications and references were provided in the manuscript.

Human research participants

Policy information about studies involving human research participants

Population characteristics	The index patient was diagnosed with de novo mPC at age 69 with high-volume prostatic adenocarcinoma, Gleason Score 9 (4+5), PSA 66.4 ng/mL, and bone metastasis. He had acquired castrate resistance after only 12 months of androgen deprivation therapy (ADT) and was subsequently treated with sequential treatments of docetaxel (8 cycles), abiraterone (3 months), and cabazitaxel (8 cycles). Following PSA progression (from 0.7 to 61.1 ng/mL within two months) from cabazitaxel therapy, paired PB and BMA were collected for HDSCA3.0 analysis before the 3rd line chemotherapy paclitaxel and carboplatin which maintained progression-free state for 2.8 months. Unfortunately, the patient passed away in 4.7 months after this progression.

Recruitment	This patient was registered under the pre-enrollment of “cabazitaxel with or without carboplatin” trial (NCT01505868). Following castrate resistance and further disease progression, paired PB and BMA samples were collected and shipped to University of Southern California within 24 hours of the collection for analysis with the HDSCA3.0 workflow.
Ethics oversight	The study was approved by the Institutional Review Board and adhered to the principles in the Declaration of Helsinki. The patient provided written informed consent prior to inclusion in the study.

Note that full information on the approval of the study protocol must also be provided in the manuscript.

Clinical data

Policy information about clinical studies

All manuscripts should comply with the ICMJE guidelines for publication of clinical research and a completed CONSORT checklist must be included with all submissions.

Clinical trial registration	This patient was registered under the pre-enrollment of “cabazitaxel with or without carboplatin” trial (NCT01505868), but failed to participate in the trial.
Study protocol	Study protocols are available in clinicaltrials.gov
Data collection	Data collection method is available in clinicaltrials.gov
Outcomes	Outcome description is available in clinicaltrials.gov
